# Hope of Recovery in Patients in the Terminal Phase of Cancer under Palliative and Hospice Care in Poland

**DOI:** 10.1155/2020/7529718

**Published:** 2020-08-19

**Authors:** Bożena Baczewska, Bogusław Block, Beata Kropornicka, Antoni Niedzielski, Maria Malm, Jacek Łukasiewicz, Krystyna Wojciechowska, Wiesław Poleszak, Agnieszka Zwolak, Marta Makara-Studzińska

**Affiliations:** ^1^Department of Internal Medicine in Nursing, Medical University, Chodźki 7 Street, 20-093 Lublin, Poland; ^2^The Pontifical University of John Paul II, Kanonicza 25 Street, 31-002 Kraków, Poland; ^3^Department of Humanities, Medical University, Staszica 4-6 Street, 20-081 Lublin, Poland; ^4^Department of Medical Informatics and Statistics with E-learning Lab, Medical University, Jaczewskiego 4 Street, 20-090 Lublin, Poland; ^5^University of Economics and Innovation, Projektowa 4 Street, 20-209 Lublin, Poland; ^6^Department of Strategy and Business Planning, Lublin University of Technology, Nadbystrzycka 38 Street, 20-618 Lublin, Poland; ^7^Department of Health Psychology, Faculty of Health Sciences, Jagiellonian University, Collegium Medicum, Kopernika 25 Street, 31-501 Kraków, Poland

## Abstract

**Introduction:**

The objective of the presented research is to characterize hope in the situational dimension, i.e., health, in the patients with cancer in the terminal phase of the disease, being treated in hospices and palliative care centers. Hope is very important for all the patients, especially for patients with cancer in various phases of the disease. Giving up on oncologic therapy and causal treatment is often associated with a transition into palliative care. When death and a loss of values become a threat, the individual has got hope to rely on. *Material and Methods*. The study relies on the Test to Measure Hope in the Health Context (NCN-36) by B.L. Block. 246 patients in the terminal phase of cancer participated in the study.

**Results:**

The internal structure of hope of recovery in the patients' group was varied. The patients showed low levels of hope of recovery since they do not believe in the effectiveness of treatment. They were also not convinced of the effectiveness of modifications in dieting, lifestyle, or the use of nonconventional medicine. They trusted the doctor in charge and were moderately satisfied with the therapy in use. The intensity of hope of recovery was on the low level in the patients in the terminal phase of cancer. Age, sex, place of living, and marital status had a significant influence on the level of hope of recovery. Variables such as living on one's own or living with one's family, socioeconomic status, education, or profession did not affect the level of hope of recovery.

**Conclusions:**

The presented results allowed as to conclude that the assessment of hope in terminally ill cancer patients can be considered as one of the important tools enabling the personalization and the improvement of palliative care.

## 1. Introduction

A proper human development is shaped by appropriate proportions between trust and mistrust. A basic sense of trust is shaped as early as the oral-sensory phase of life [1]. It manifests itself in infants being able to sleep safely, take in food, and excrete. Experiencing various situations in life, accompanied by feeling safe and close to the mother, leads to a sense of togetherness, intimacy, and trust, even when the mother is not there. Trust and self-confidence remain, since they were formed on the basis of fixed situations repeated at the same time of the day. Hence, the child learns how to trust the mother, then adults, and then himself or herself. Such self-confidence must take over mistrust and is needed for the child's healthy development. A correct sense of balance between trust and mistrust leads to the emergence of hope. Hope is the earliest and indispensable virtue which accompanies us throughout our lives [2].

Each illness and disease disturbs our rhythm of life. They cause anxiety, irritability, and even anger. Patients experience fear and uncertainty [3]. Erikson claims that “hope is the persistent conviction in a chance to fulfill desires, in contrast to negative impulses and wild emotions, which are characteristic of the early phases of human existence” [2]. Zavalloni describes hope as an actual drive behind human activity, as a symptom of life. He talks of hope beyond rationalism, hope within individuals, and hope beyond them [1]. When the conditions in life are life-threatening and core values are in danger, hope appears to be of importance. It becomes a faithful and desired companion of the lost, defeated, hurt, hungry, weak, and unhappy ones [1].

Experiencing a disease is a great weight for the patient. The healthy and the sick often consider the phase of being ill as a phase of passivity, inertia, of being valued as useless and unproductive [3]. Hope accompanies all patients, yet it is of special importance to those suffering from cancer, at various stages of the disease. Giving up on aggressive oncological treatment and causal treatment goes together with an offer to be subject to palliative care. It is a great challenge for the patients. They need to accept the fact of being terminally ill, which is difficult in the case of harbouring great hope of recovery. Losing this hope should give way to another type of hope, i.e., for suitable, dignified care. It is a difficult moment not only for the doctors, but also for the patients' family. So far, all the activity was geared to motivate the patients to fight the disease. It takes courage to inform the patients that treatment is ineffective and does more harm than good. When there is a necessity to end medical treatment, the patients need to be informed about it, should be provided with a plan for further medical care, and should receive support.

Around the world, there are many researchers who studied the hope of cancer patients, such as Nowotny [4], Cohen et al. [5], Nekolaichuk [6], Frankl [7], and Scioli and Biller [8], who performed similar studies.

### 1.1. Objectives

The objective of this study is to describe hope in the situational context, i.e., health of the patients suffering from terminal cancer, located in hospices and palliative care centers.

The particular goals of the study are as follows:

1. Characteristics of the internal structure of hope of recovery in patients suffering from terminal cancer
2. Determining the level of hope of recovery in patients suffering from terminal cancer
3. Determining the correlations between hope of recovery and sociodemographic variables

## 2. Materials and Methods

The study was performed by the team supervised by Bogusław Block in 2010-2016 in Poland and approved by the Bioethics Committee of the Medical University of Lublin (decision no: KE-0254/225/2010).

The selection criterion was the patients' consent, the diagnosis of terminal cancer, and the patients staying in a hospice or palliative care center.

The study was performed using the method of standardized interview. Each session with the patient was not only of a diagnostic but also of a therapeutic character. The atmosphere during the interviews was friendly. The interviewees could ask for clarifications if the questions were incomprehensible. Anonymity of the interviewees was preserved. The tool used in the study was Test for Measuring Hope in the Health Context (NCN-36), designed by B.L. Block specially for patients with diagnosed cancer [9]. The test consists of 4 scales isolated by means of the factor analysis (8 items and 4 buffer questions in each scale): (1) health—situational dimension, (2) objectives—telic-temporal dimension, (3) religious beliefs—spiritual dimension, and (4) motivation—emotional-affective dimension. The Cronbach's alpha rate for the test was 0.92, and the rate for its particular scales ranged between 0.72 and 0.86.

Interpretation of the scores was done on the basis of the mean scores, which ranged between 1 and 7. The results of 6.0-7.0 range point towards a strong level of hope, 5.0-5.99 range towards a moderate level of hope, 3.0-3.99 towards a low level of hope, 2.0-2.99 towards a very low level of hope, and 1.0-1.99 towards hopelessness. The scale of hope experienced by the patients is reflected by their global results. The profile of hope is shaped through the results of each particular scale.

This study presents and comments on detailed scores within the situational dimension of hope (health), both from a global perspective and in the context of particular items.

### 2.1. Statistical Analysis

The statistical analysis offers results in group tables (correlation tables), in the percentage form. The statistical analysis for qualitative features expressed in nominal scales was based on Pearson's chi-squared independence test. For qualitative features with a regulation potential (expressed in Likert's scale), nonparametric tests were used. In order to make comparisons between two groups of codependent variables, the Wilcoxon rank test was used, and in order to make comparisons between more than two groups, the ANOVA Friedman test was used. For independent qualitative features, median test was used. For independent qualitative features with a regulation potential and for quantitative features in order to make comparisons between two groups, the Mann-Whitney *U* test was used (independent variables). In order to make comparisons between more than three variables, median test and the Kruskal-Wallis test were used.

## 3. Results

The research was carried out among 246 patients in the terminal phase of a neoplastic disease. The subjects were patients of hospice and palliative care units, located in 17 day care centres and 24-hour facilities throughout Poland. The average age of the respondents was 59.5; the youngest was 18, and the oldest was 90. The most numerous group comprised people aged between 51 and 65—99 subjects, which constitutes 40.24% of all respondents. The smallest group of respondents comprised of people up to 35 years of age (5.69%). People over 65 years of age constituted 34.96% of the researched group, and 47 patients were in the age range of 36–50 years (19.11%).

Most of the respondents were female—150 (60.98%), and the prevailing number of people lived in cities and towns—192 (78.05%), while most of the respondents lived together with their families—155 (60.01%), 20 (8.13%) lived with nonrelatives, and the remaining group—71 (28.86%)—lived alone.

Most of the respondents were married—117 (47.56%). The second biggest group comprised widowed respondents—67 (27.24%), and the third biggest were the divorced—32 (13.01%). Unmarried people constituted the smallest group of respondents—30 (12.20%). Every third respondent completed secondary education—82 people (33.33%), and there were two equally numerous groups of patients who completed primary and vocational education—59 (24.22%) respondents in each of these groups. People with higher education constituted 11.79% (29 respondents), and there were 17 (6.91%) patients with incomplete higher education or holding a bachelor's degree.

Most often, the subjects assessed their financial situation as satisfactory—111 (45.12%) patients, and nearly 1/3 of the respondents described it as bad—73 (29.67%). Only every fourth respondent perceived it as good—52 (21.14%) or very good—10 (4.07%).

Tested items comprising the situational dimension measured hope in the health context in the following areas: (a) treatment by a trusted doctor of conventional medicine, (b) following an appropriate diet and lifestyle, (c) nonrational feelings for recovery, (d) trust in nonconventional medicine.

### 3.1. Distribution of Levels of Hope in Patients

The study shows the levels of hope in the health dimension, on the basis of the interview with the patients.  Table 1 shows the results obtained in particular items that contributed to the situational dimension of hope.

Belief in recovery was observed in 40.35% of the responses (where 12.35% concerned a strong belief, 13.36% a strong belief, 14.63% a weak belief). A lack of hope of recovery was observed in 46.34% of the responses (15.65% were highly negative, 15.60% negative, and 15.09% rather negative). 13.31% of the respondents did not have any opinion on the issue. Distribution of the responses differed significantly for *p* ≤ 0.001.

### 3.2. Hope of Recovery Found in Appropriate Treatment Supervised by a Trusted Physician of Conventional Medicine (a Area)

Almost every third respondent (32.12%) hoped that the treatment will lead to a full recovery, even though the intensity of the hope varied. More than a half of the respondents (56.1%) had some serious doubts when it came to the effectiveness of the treatment. 11.79% of the respondents were not able or did not want to comment on the effectiveness of the treatment.

More than half of the respondents (52.45%) were not convinced that the available medicine is equipped with tools capable of treating a cancer successfully. In this group, 17.89% of the respondents did not believe in effectiveness of their treatment. Exactly the same percent of the respondents did not have any hope in this respect; 16.67% were not convinced.

51.63% of the respondents were satisfied with the treatment, and the intensity of the satisfaction varied. 28.45% of the respondents were disappointed with the treatment. Every fifth (19.92%) respondent did not want to comment on the issue.

The majority of the respondents (71.95%) trusted the physician in charge, even though the levels of trust varied. The rest of the respondents did not trust their physicians (14.23%) or did not comment on the issue (13.82%).

### 3.3. Hope of Recovery Found in a Healthy Diet and a Lifestyle (b Area)

Our study shows that 36.58% of those suffering from cancer state that a healthy diet and lifestyle can contribute to a full recovery. It was a conviction of every tenth respondent, and every eighth respondent believed it was possible. A neutral stance was adopted by 13.82% of the interviewees. They maintained that a healthy diet and lifestyle will not be beneficial or detrimental to their health situation. Almost half of the respondents (49.59%) did not believe that a healthy diet and lifestyle will lead to a recovery (within the group, 16.26% did not believe it all, 17.48% rather did not believe it, and 15.85% definitely did not believe it).

### 3.4. Hope regarding Recovery Intuition (c Area)

Consciousness of the seriousness of one's illness is not equal to a loss of hope of a full recovery. Such a belief was found in 30.48% of the respondents. The intensity of the belief was evenly distributed. 11.38% of the respondents did not have an opinion on the issue. The majority (58.14%) of those suffering from cancer, being aware of their serious health state, lost their hope of recovery. It does not mean, however, that the situation was of a static character and could not change in the future.

Our study showed that, among those in the terminal phase of cancer, the intuition regarding recovery varied. A third of the respondents (34.56%) experienced such an intuition. Yet the majority (53.66%) of those suffering from cancer had a feeling that they would never recover. 11.79% of the respondents claimed that they did not experience any negative or positive feelings in this regard. Possibly, these respondents distanced themselves from their health existential situation and did not want to focus on their intuition. According to Tischner: “Our hope is such as our participation. When we »get out« of participation, we lose hope” [10].

### 3.5. Hope in Nonconventional Medicine (d Area)

Our study shows that the majority of those suffering from cancer (58.13%) did not believe in the effectiveness of nonconventional medicine. Every third respondent believed in nonconventional medicine. Within this group, the biggest subgroup was formed by those with a weak trust in nonconventional medicine (13.36%), and the smallest group (12.35%) had a strong belief in nonconventional medicine. 13.01% did not take any stance on the issue.

### 3.6. Intensity of Hope on the Basis of Arithmetic Mean Results

The obtained results show the intensity of hope on the basis of arithmetic mean results, which are presented in Table 2.

The level of hope in the group of patients in the terminal phase of cancer, in the situaitonal dimension—health, reached the value of 3.88, which is low. The patients in the group did not form a homogeneous group when it comes to the levels of hope. The patients demonstrated a high level of hope (5.28) only when it comes to trust in the physicians in charge. A moderate level of hope was observed (4.45) when it comes to the satisfaction with the treatment. A low level of hope of recovery was observed regarding a healthy diet and lifestyle (3.73), effectiveness of contemporary medicine (3.68), effectiveness of the treatment (3.44), nonconventional medicine (3.22), recovery intuition (3.59), and hope of recovery (3.42).

### 3.7. Levels of Hope in the Situational Dimension

The results in the health dimension were distributed unevenly on six levels, from the level of hopelessness to the level of a very strong hope. They are presented in Table 3.

The presented data show that every fourth respondent (25.20%) experienced hope on the moderate level and on the strong level (24.80%). A low or very low level of hope was observed in 1/3 of the patients (34.96%). The feeling of hopelessness was experienced by 7.72%. Similarly, a very strong level of hope was observed in 7.32% of the patients.

### 3.8. Correlations between Sociodemographic Data and Hope

We wanted to check if there are correlations between hope in the situational dimension—health and age, gender, place of living, marital status, education, financial situation, and socioeconomic status. Correlations were found between the subject of hope and the general results and age, gender, place of living, and marital status. The detailed data are presented in Table 4.

The variable of age did not affect the level of hope in the group of patients suffering from cancer. We found negative correlation factors for the following items: hope of recovery, intuition regarding recovery, and belief in nonconventional medicine. This means that the younger the patient, the higher level of hope of recovery they demonstrate.

When it comes to the gender of the patients, a statistically higher level of hope in the global situational dimension was observed in women (*p* = 0.018) rather than men. Women had higher levels of hope regarding effectiveness of treatment, medicine's capability of successful treatment and recovery.

The place of living did not correlate with the level of hope. It had a correlation with trust in the physicians in charge. Those living in rural areas trusted their physicians more than those living in urban areas (*p* = 0.016).

Marital status did not affect the level of hope. Those who were married had higher levels of hope regarding effectiveness of nonconventional medicine (*p* = 0.047).

No correlation was found between variables such as place of living, socioeconomic status, education or financial situation, and levels of hope.

In the global situational dimension, higher levels of hope were observed in women.

## 4. Discussion

The results presented in this paper are the part of the comprehensive studies concerning the multifaceted assessment of hope among the terminally ill cancer patients undergoing paliative and hospice care in Poland. The preliminary observations concerning the hope vs. place of dwelling were published by Baczewska et al. [11]. The objective of the presented research is to characterize hope in the situational dimension, i.e., health, in the patients with cancer in the terminal phase of the disease, being treated in hospices and palliative care centers [11].

Hope for recovery is not expected in the terminal phase of cancer. Kűbler-Ross claims that the last stage of dealing with the illness is acceptance of one's imminent death. This means that the patients accepted they cannot be cured. However, patients may withdraw to the cancer's earlier stages or fixate on one of them for some period of time and sometimes show hope for recovery until they die [12].

Even though, objectively, the situation of the patients in the terminal phase of cancer entails an imminent death, almost 92% of the patients in our study showed a stronger or weaker level of hope of recovery. Only 7.72% of the patients experienced a feeling of hopelessness. It can be assumed then that those who lost hope of recovery take a realistic perspective on their health situation. They probably accept the imminence of death, the last stage of dying. The character of hope of the patients in the terminal phase of cancer is dynamic. Some patients, devoid of hope of recovery, may experience a breakdown related to their poor health.

Almost as numerous as the group who lost hope of recovery was the group who experienced strong levels of hope (7.32%). This group does not accept the perspective of imminent death. They reject such a perspective and negate it, which is a strong self-defence mechanism. The patients do not take the seriousness of their situation into consideration. Every other patient demonstrated a strong or moderate level of hope, which shows that they did not accept the terminal phase of the illness. They still believed they could recover. The other terminally ill patients were close to coming to terms with the imminence of death caused by cancer.

This study shows a correlation between gender and the global levels of hope of recovery, and also between some items and age, gender, place of living, and marital status.

When it comes to the age of the patients, a higher level of hope was observed in women rather than in men (*p* = 0.018) in the global situational dimension—health. The same conclusion was supported by a study performed by Block [9], where women show a high level of hope while men show a moderate level of hope, and the differences are statistically significant (*p* ≤ 0.001).

Age did not correlate with intensity of hope in the group of patients in the terminal phase of cancer. A statistical analysis showed that age correlates negatively and significantly with three items. This means that hope of recovery diminishes with age. According to Block, higher levels of hope are experienced by those in the productive age and in young people below 35 years of age rather than in the retired group [9]. Younger people's hope of recovery, intuition regarding recovery, and belief in effectiveness of nonconventional medicine played a role for the intensity of hope of recovery. Young people come to terms with death with more difficulty. They also have more difficulty with moving forward to the stage of death acceptance [12] and have more difficulty discussing negative prognosis.

The most common factor concerning hope in the studied group was trust in the physicians in charge. The mean results show that the patients experienced a strong level of hope (5.28) when it came to trust in the physicians in charge. The majority of the patients (71.95%) trusted their physician in charge, and it was rather the patients living in rural rather than urban areas. According to Grynchutskyi and Machuga [13], an appropriate standard of health service is translated into the patients' health, security, life, and trust in the physicians. According to the study carried out in the Leicestershire Cancer Center in Great Britain, the level of trust in the physician in charge was on the 81.4% level in British South Asian patients and 78% in British White patients [14].

The study performed by Eliott and Olver [15] shows that hope of recovery was commonly observed in the group of 28 patients suffering from cancer. The hope is perceived as indispensable for life and has a dynamic character. Some researchers suggest that the patients may experience hope of recovery while simultaneously accepting the ultimate character of the illness [16]. Our study shows that 41.86% of the patients, while being aware of the seriousness of their health condition, did not lose hope of recovery. 46.34% of the patients had a feeling they would recover. This begs a question what makes the patients build their hope, even in the face of illusory premises. Perhaps it is done due to the sixth sense, located in the forebrain [17]. The sixth sense gives us solutions out of emotionally complicated situations, or when faced with an important choice or dilemma. It is based on the knowledge we collected throughout our lives. According to Damásio, intuitive feelings are the results of an unconscious accumulation of experiences, the information of which are registered in the neural paths of the brain, in the so-called prefrontal cortex. It registers all the events in which we participated, no matter how trivial or seemingly not important. Even though the register is kept for life, our consciousness does not have access to most of the information. Perhaps the experience gathered by the patients in our study pointed to a possibility of a way out of every difficult situation [17].

Health is contingent on many factors. They were described by Lalonde [18] and function in the literature as the so-called health fields. Health is dependent mostly on individuals, i.e., their lifestyle, comprising physical activity and eating habits (53%). However, in the case of patients in the terminal phase of cancer, only 3.73% hoped for recovery in relation to a healthy diet and lifestyle, and 49.59% did not believe that a healthy diet and lifestyle can be beneficial for their health condition.

Nonconventional medicine still remains a controversial issue. The methods of treatment belonging to this area are not verified scientifically. Some academic studies point towards their ineffectiveness. Składowski et al. [19] showed that every other patients suffering from cancer tried alternative and nonconventional methods of treatment. The reason for this tendency is a fear of cancer, of a demanding oncological treatment, of death, limited means of conventional medicine, faith in nature, and inefficient health system which does not meet all the needs and expectations of the patients. The initiators of paramedical activity are often the dearest and nearest of the patients.

A turn towards natural methods of treatment can be understandable in the case of those patients for whom conventional medicine failed. These patients, and their families, seek hope and help in hopeless cases. Nonconventional methods of cancer treatment substitute traditional treatment when the latter does not bring any results. However, it may be dangerous when nonconventional treatment replaces well-founded and recommendable forms of treatment. Our study shows that 58.13% of the patients did not believe that nonconventional medicine can be effective. Interestingly, 52.45% of the patients did not believe in the effectiveness of conventional medicine. Those patients who were married were statistically more often convinced that nonconventional medicine can be effective.

Referring back to the study performed by Kűbler-Ross [12], we may state the hope can be found on all levels in the group of patients in the terminal phase of cancer, is characterised with great dynamics, and is dependent on many variables. Hope of recovery in the patients aware of the imminent death is a complex and personal phenomenon, which requires individual strategies of support [20].

## 5. Conclusions

1. The internal structure of hope of recovery in the patients in the terminal phase of cancer is diverse. The patients showed low levels of hope of recovery since they believe that medicine is not equipped with effective tools of treatment. They believe that conventional treatment is ineffective, just as is a healthy diet and lifestyle, or the use of nonconventional forms of treatment.

The patients showed high levels of trust in their physicians in charge and were moderately satisfied with the treatment they received.

(2) The level of hope of recovery in the patients in the terminal phase of cancer was low
(3) Age, gender, place of living, and marital status were positively correlated with the level of hope of recovery
(4) Negative correlation was found between the level of hope of recovery and variables such as living on one's own or with a family, socioeconomic status, education, financial situation

### 5.1. Limitations

Participants were patients of hospice and palliative care units only from Poland. The selection criterion for the present research was the age at least of 18 and agreement to participate in the study before its beginning.

## Figures and Tables

**Table 1:** Distribution of levels of hope in the situational dimension—health (*N* = 246).

Test item	Distribution of frequency (*N*, %)							
	Areas	Positive beliefs			No opinion	Negative beliefs		
		Definitely yes	Yes	Rather no	I do not know	Rather no	No	Definitely no
1. Belief in the effectiveness of the treatment	a	21, 8.54%	21, 8.54%	37, 15.04%	29, 11.79%	47, 19.11%	40, 16.26%	51, 20.73%
2. Belief in contemporary medicine	a	32, 13.01%	26, 10.57%	32, 13.01%	27, 10.98%	44, 17.89%	41, 16.67%	44, 17.89%
3. Satisfaction with the treatment	a	30, 12.20%	44, 17.89%	53, 21.54%	49, 19.92%	36, 14.63%	17, 6.91%	17, 6.91%
4. Hope of recovery with a recognition of the seriousness of the illness	c	25, 10.16%	25, 10.16%	25, 10.16%	28, 11.38%	43, 17.48%	50, 20.33%	50, 20.33%
5. Belief in the effectiveness of an appropriate diet and lifestyle.	b	25, 10.16%	34, 13.82%	31, 12.60%	34, 13.82%	43, 17.48%	40, 16.26%	39, 15.85%
6. Trust in the physicians in charge	a	65, 26.42%	64, 26.02%	48, 19.51%	34, 13.82%	19, 7.72%	10, 4.07%	6, 2.44%
7. Intuition regarding recovery	c	27, 10.98%	29, 11.79%	29, 11.79%	29, 11.79%	40, 16.26%	48, 19.51%	44, 17.89%
8. Belief in nonconventional treatment	d	18, 7.32%	20, 8.13%	33, 13.41%	32, 13.01%	25, 10.16%	61, 24.80%	57, 23.17%
Hope regarding health	abcd	243, 12.35%	263, 13.36%	288, 14.63%	262, 13.31%	297, 15.09%	307, 15.60%	308, 15.65%
		794, 40.35%			262, 13.31%	912, 46.34%		

ANOVA Friedman test: Chi^2^ ANOVA (*N* = 246, df = 7) = 286.1424; *p* ≤ 0.001; a: treatment by a trusted doctor of conventional medicine; b: following an appropriate diet and lifestyle; c: nonrational feelings for recovery; d: trust in nonconventional medicine.

**Table 2:** Descriptive statistics for the levels of hope in the situational dimension—health (*N* = 246).

No.	The subject of hope	Descriptive statistics					
		Mean, *M*	Trust, -95%	Trust, 95%	Median, Me	Modal, Mo	Standard deviation, SD
1	Belief in the effectiveness of the treatment	3.44	3.20	3.68	3	1	1.91
2	Belief in contemporary medicine	3.68	3.43	3.94	3	Multiple	2.02
3	Satisfaction with the treatment	4.45	4.23	4.66	5	5	1.70
4	Hope of recovery with a recognition of the seriousness of the illness	3.42	3.17	3.67	3	Multiple	1.98
5	Belief in the effectiveness of an appropriate diet and lifestyle.	3.73	3.49	3.98	4	3	1.95
6	Trust in the physicians in charge	5.28	5.08	5.47	6	7	1.56
7	Intuition regarding recovery	3.59	3.34	3.84	3	2	2.00
8	Belief in nonconventional treatment	3.22	2.98	3.47	3	2	1.93
Global	Hope of recovery	3.88	3.71	4.05	3.63	3.5	1.36

**Table 3:** Distribution of the results of hope in the situational dimension—health.

Results	Table of quantity values—health dimension			
	Number	Accumulated number	Percent	Accumulated percent
1.0-1.99	19	19	7.72	7.72
2.00-2.99	37	56	15.04	22.76
3.00-3.99	49	105	19.92	42.68
4.00-4.99	62	167	25.20	67.88
5.00-5.99	61	228	24.80	92.68
6.00-7.00	18	246	7.32	100.00

**Table 4:** Sociodemographic variables which show statistically significant codependencies regarding hope in the context of health.

Subject of hope	Sociodemographic variables			
	Age^1^	Gender^2^	Place of living^3^	Marital status^4^
Belief in the effectiveness of the treatment	*p* = 0.123 (*R* = −0.099)	*p* = 0.182	*p* = 0.137	*p* = 0.334
Belief in contemporary medicine	*p* = 0.742 (*R* = −0.02)	*p* = 0.042	*p* = 0.059	*p* = 0.905
Satisfaction with the treatment	*p* = 0.240 (*R* = −0.075)	*p* = 0.004	*p* = 0.980	*p* = 0.685
Hope of recovery with a recognition of the seriousness of the illness	*p* = 0.018 (*R* = −0.150)	*p* = 0.003	*p* = 0.078	*p* = 0.572
Belief in the effectiveness of an appropriate diet and lifestyle.	*p* = 0.579 (*R* = −0.036)	*p* = 0.211	*p* = 0.309	*p* = 0.281
Trust in the physicians in charge	*p* = 0.478 (*R* = −0.045)	*p* = 0.113	*p* = 0.016	*p* = 0.128
Intuition regarding recovery	*p* = 0.038 (*R* = −0.132)	*p* = 0.122	*p* = 0.433	*p* = 0.922
Belief in nonconventional treatment	*p* = 0.023 (*R* = −0.145)	*p* = 0.096	*p* = 0.328	*p* = 0.047
Hope of recovery	*p* = 0.070 (*R* = −0.116)	*p* = 0.009	*p* = 0.280	*p* = 0.887

Analysis based on ^1^Spearman's coefficient, ^2^Mann-Whitney *U* test, ^3^Median test, and ^4^Kruskal-Wallis test.

## Data Availability

The data that support the findings of this study are available on request from the corresponding author. The data are not publicly available due to privacy or ethical restrictions.
